# A Rare Case Presentation: Intravesical Catheter Knotting

**DOI:** 10.1089/cren.2018.0016

**Published:** 2018-06-01

**Authors:** Enis Kervancioglu

**Affiliations:** Department of Urology, Baskent University School of Medicine, Ankara, Turkey.

**Keywords:** voiding cystourethrography, urethral catheterization, knotting

## Abstract

Urethral catheterization is a common invasive procedure applied for several purposes on children. Voiding cystourethrogram (VCUG) is a diagnostic fluoroscopic procedure, which is commonly used in assessing urinary tract infections in children and which requires urethral catheterization. We report a case of intravesical catheter knotting, which is a rare complication of VCUG.

## Introduction

Urethral catheterization, which is commonly used in the clinics for various purposes such as urine sample collection, monitoring of fluid therapy, clean intermittent catheterization, voiding cystourethrogram (VCUG), is an invasive procedure and has a low complication rate. Infant feeding tubes or Foley catheters are mainly used on infants and children for urethral catheterization purposes. VCUG is a diagnostic method often used for urinary tract infection assessment on children. Related complications are very rare and some of the reported complications are infection, catheter-associated trauma, bladder rupture and contrast-induced allergic reactions.^1^ Urethral catheterization is required for VCUG. Knotting of the feeding and Foley catheters inserted into the bladder through urethral catheterization is a very rare complication.^2^ It may clinically be manifested as the inability to deflate the catheter balloon or the inability to pull out the balloon-free catheter, and as such, any attempt to withdraw the catheter together with the knot may result in urethral injury. An attempt to withdraw the catheter that is not a full knot might result in the knot becoming even tighter. Should such a knotting be identified, it is possible to remove it through a surgical operation or even other simpler techniques. If the said knot is somewhat loose, a guidewire along with flouroscopy might help resolve the issue. Attempting to remove a catheter with a tight knot, however, requires a surgical operation through cystotomy or cystoscopy.

## Case Presentation

**FIG. 1.**
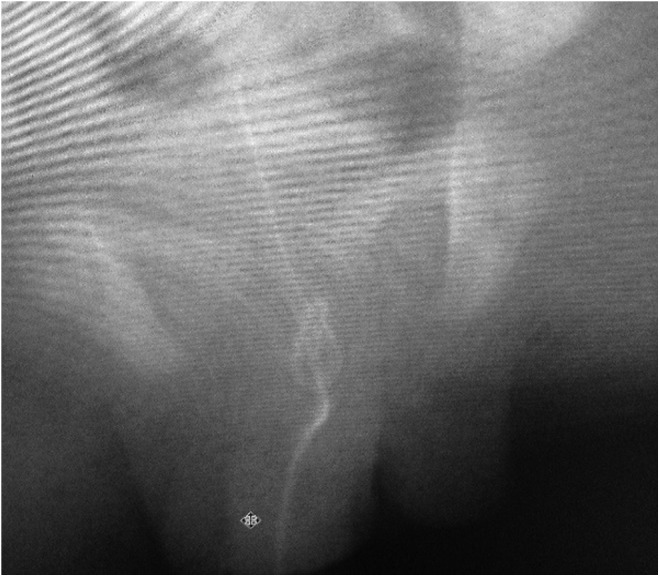
Introducing a guidewire through the catheter under fluoroscopy guidance and imaging of the knot.

**FIG. 2.**
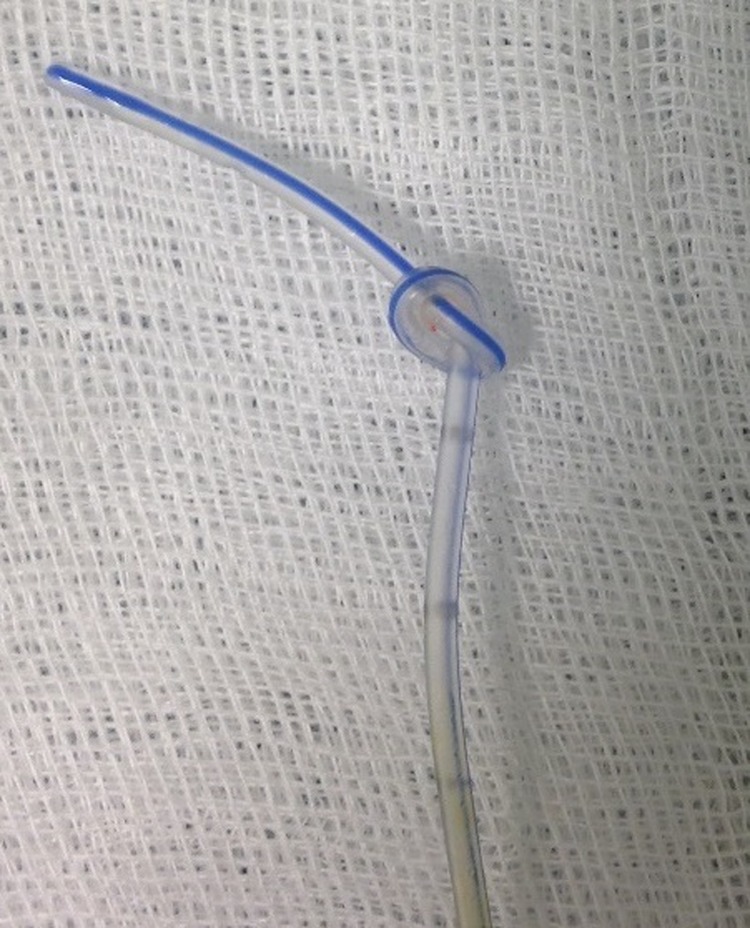
Knotted catheter.

A 19-day-old male infant was urethral catheterized with a 6F infant feeding tube followed by a VCUG for evaluation of vesicoureteral reflux. After the procedure, the catheter could not be removed. Therefore, the pediatric unit consulted us regarding the problem. Under fluoroscopic guidance, a guidewire was inserted through the feeding tube to uncoil it, but this failed (Fig. 1). Therefore, we planned to cut the knot endoscopically under general anesthesia. The patient was taken to the operating room and put under general anesthesia. Considering the relaxation effect of the anesthesia before cystoscopy and the danger of urethral trauma, it was then planned to remove the catheter by gently pulling it out. Since there was no resistance encountered and no serious tension on the catheter was observed, the operation proceeded as planned. The catheter seemed to be removed with ease. There are cases in the literature that report of removing knotted catheters using the said method.^2^ After the catheter was removed fully with the knot at the tip (Fig. 2), cystoscopy was performed to check for any potential urethral injury. Mucosal integrity of the urethra was observed to be intact and the patient was discharged after a short follow-up and observing spontaneous micturition.

## Discussion

Urinary tract catheterization is used for both diagnostic and therapeutic purposes among the pediatric age group. One of the most common uses for diagnostic purposes is VCUG.^2^ Intravesical catheter knotting is mostly reported in the patient age group of 2 years and lower.^3^ Incidence of urinary catheter knotting is reported to be 0.2 per 100,000 catheterizations.^2^ The knotting most likely occurs because of excessive insertion of the small caliber catheter inside the bladder. Catheters inserted excessively as such lead to a formation of a loop inside the bladder. During contraction of the bladder, the end of the catheter coils on this loop and thus makes a knot, which is not yet tight.^3^ Any attempt to remove that catheter tightens the knot further. It is very important to identify this complication beforehand. Although radiologic images can help identify the knots, we should keep in mind such complications should we experience difficulties while removing catheters.

Several methods are available to remove a knotted catheter. These methods are continuous gentle pulling with general anesthesia, cystotomy, or removal by endoscopy. The most common method is gentle but continuous retraction under the effect of general anesthesia; however, this method may result in development of a urethral trauma and later urethral stricture.^2^

It is possible to prevent urethral catheter knotting through careful selection of the catheter and a better understanding of the urethral anatomy and safe insertion lengths.^2^ A newborn male urethra measures 5 cm; this increases to 8 cm at the age of 3 years and 17 cm at adulthood. In females, urethral length is relatively smaller and grows at a slower rate. Female urethra measures 2.18 cm at birth and increases to 2.54 cm in 5 years and 3.78 cm at adulthood.^2^ The part inserted from the urethra should be kept as short as possible, sizing should be made beforehand, and the catheter should not be inserted further after observing urine flow.^4^ Use of very flexible feeding tubes should be avoided as much as possible.^4^ Other measures to be taken are removal of the catheter as early as possible, or if the catheter has to be left in place for fluid monitoring, safely and tightly securing the catheter to prevent any further insertion, and gentle yet continuous pulling of the catheter during the removal procedure.^4^

In conclusion, indications for urethral catheterization must be carefully considered since it is an invasive procedure commonly applied in the clinics for various purposes. Although intravesical catheter knotting is a rare complication, it can easily be prevented by taking simple measures.

## Abbreviation Used

VCUG: voiding cystourethrogram
